# Factors associated with severity in *Klebsiella pneumoniae* community-acquired pneumonia: A retrospective cohort study from South India

**DOI:** 10.1016/j.nmni.2026.101741

**Published:** 2026-03-23

**Authors:** Souvik Chaudhuri, Tirlangi Praveen Kumar, Shreya Das Adhikari, Danavath Nagendra, Prithvishree Ravindra, Rachana Bhat, K.E. Vandana, Nitin Gupta

**Affiliations:** a Department of Critical Care Medicine, Kasturba Medical College, Manipal Academy of Higher Education, Manipal, 576104, India; b Department of Infectious Disease, Kasturba Medical College, Manipal Academy of Higher Education, Manipal, 576104, India; c Department of Anaesthesiology, Kasturba Medical College, Manipal Academy of Higher Education, Manipal, 576104, India; d Department of Emergency Medicine, Kasturba Medical College, Manipal Academy of Higher Education, Manipal, 576104, India; e Department of Microbiology, Kasturba Medical College, Manipal Academy of Higher Education, Manipal, 576104, India

**Keywords:** *Klebsiella pneumoniae*, Community-acquired pneumonia, Antimicrobial resistance, Predictors of severity, ICU admission

## Abstract

**Problem considered:**

*Klebsiella pneumoniae* is increasingly recognized as a pathogen of concern causing community-acquired pneumonia (CAP) due to its association with diabetes, alcohol use, and antimicrobial resistance. However, data from India regarding severity in *K. pneumoniae* CAP is limited. The primary objective of this study was to determine the factors associated with severity in *K. pneumoniae* CAP.

**Methods:**

We conducted a retrospective cohort study of culture-confirmed *K. pneumoniae* CAP at a tertiary care hospital between January 2017 and December 2023. Eligible cases were defined as those with radiological evidence of CAP and culture positivity from respiratory or blood samples obtained within 48 h of admission. Demographic, clinical, laboratory, radiological, microbiological, treatment, and outcome data were extracted. Factors associated with severity were identified using multivariable logistic regression.

**Results:**

Of 277 culture-confirmed cases of *K. pneumoniae* from samples sent within 48 h of admission, 105 fulfilled inclusion criteria. Of these, 32 patients (30.5%) had a severe disease. Patients with severe disease frequently reported alcohol use, presented with bacteraemia, shock, higher leukocyte counts, elevated urea, lower serum albumin, and greater antimicrobial resistance. On multivariable analysis, factors associated with severe disease were alcohol use (aOR 18.8, 95% CI 3.5–101), bacteraemia (aOR 11.8, 95% CI 1.8–77.5), ceftriaxone resistance (aOR 6.6, 95% CI 2.1–20.5), and higher leukocyte counts (aOR 1.15 per unit increase, 95% CI 1.05–1.26).

**Conclusion:**

*K. pneumoniae* isolates from patients with CAP are frequently resistant to ceftriaxone. Ceftriaxone resistance, along with alcohol use, bacteraemia, and high leukocyte count, was associated with severe disease.

## 1 Introduction

Community-acquired pneumonia (CAP) is a leading infectious cause of morbidity and mortality worldwide, accounting for millions of hospitalizations annually and posing a substantial economic and health care burden [1,2]. The epidemiology of CAP varies geographically, influenced by host factors, comorbidities, and local microbiological ecology. In India, CAP represents a significant public health problem, with high rates of hospitalization and case fatality, especially among older adults and those with comorbid illnesses [1,2]. Traditionally, *Streptococcus pneumoniae* has been regarded as the most common bacterial cause of CAP. However, in recent decades, Gram-negative organisms have gained prominence in many regions, particularly in Asia [[2], [3], [4], [5]]. *Klebsiella pneumoniae* stands out as an important pathogen due to its dual clinical and microbiological challenges [6,7]. Clinically, it is frequently associated with host risk factors such as diabetes mellitus, chronic alcohol use, and underlying immunosuppression [6,7]. Microbiologically, it is increasingly recognized for its potential to harbour antimicrobial resistance mechanisms, including extended-spectrum β-lactamase and carbapenemase production [6,7].

Of particular concern is the emergence of resistant *K. pneumoniae* lineages across Asia, which cause severe invasive syndromes [6,7]. These strains are associated with poor outcomes and limited therapeutic options, raising alarms for clinicians and public health authorities [6,7]. Despite this clinical significance, systematic data on the epidemiology, antimicrobial resistance patterns, and outcomes of *K. pneumoniae* CAP in India are scarce. Empiric treatment guidelines for CAP in India, as in many other countries, generally recommend a third-generation cephalosporin such as ceftriaxone in combination with either azithromycin or doxycycline [8]. This regimen provides broad coverage for classical respiratory pathogens, particularly *S. pneumoniae*, which remains largely susceptible to ceftriaxone [2]. However, such empirical choices may be inadequate for *K. pneumoniae*, especially given the rising prevalence of ceftriaxone resistance. Undertreatment in these cases risks clinical deterioration, septic shock, and death.

Given the growing importance of *K. pneumoniae* in CAP and the paucity of Indian data, there is an urgent need to better characterize the clinical features, resistance profiles, and outcomes of affected patients. Therefore, we conducted a retrospective cohort study of culture-confirmed *K. pneumoniae* CAP at a tertiary care hospital in South India to identify factors associated with severe disease.

## 2 Methods

*Study design and study setting:* This retrospective cohort study was conducted at Kasturba Medical College Hospital, Manipal, a large 2032-bedded tertiary care referral centre in coastal South India. The hospital serves patients from several surrounding districts and offers advanced diagnostic services, including microbiology, radiology, and intensive care. The study period extended from January 2017 to December 2023. Approval was obtained from the Institutional Ethics Committee, and informed consent was waived owing to the retrospective design and the exclusive use of anonymized data.

*Study participants*: Patients with CAP, defined as a new radiological infiltrate on chest radiograph, together with at least two clinical features: fever (≥38 °C), cough, or dyspnoea, with a symptom duration of less than 14 days. Severity of illness was defined pragmatically as the need for CAP patients to be admitted to the intensive care unit (ICU) during hospitalization.

*Inclusion criteria:* CAP patients with culture-confirmed *K. pneumoniae* infection, isolated from respiratory specimens such as sputum, bronchoalveolar lavage, or endotracheal aspirates, or blood cultures obtained within 48 h of admission. Only monomicrobial infections were included in the analysis to avoid confounding attribution of outcomes.

*Exclusion criteria:* Patients were excluded if they had symptoms for more than 14 days, had no radiological evidence of pneumonia, or developed pneumonia more than 48 h after admission (to exclude hospital-acquired infections). Patients hospitalized within the preceding 14 days were excluded to avoid including hospital-acquired pneumonia cases, as were those with polymicrobial respiratory cultures. These criteria ensured that the cohort represented true community-acquired monomicrobial *K. pneumoniae* infections.

*Primary outcome:* Severe CAP requiring admission to ICU.

*Sample size:* Using the events-per-variable (EPV) approach outlined by Riley et al., we considered a final model with four predictors and aimed to achieve 10 events per variable, requiring 40 events in total [9]. With an expected prevalence of 46%, this corresponds to a minimum of 87 participants to obtain the necessary number of events [10]. To account for an anticipated 15% loss due to missingness, the calculated sample size was inflated, resulting in a final requirement of approximately 102 participants.

Data were extracted from electronic medical records using a structured proforma. Information was collected on patient demographics and comorbidities, including diabetes mellitus, chronic kidney disease, chronic liver disease, chronic lung disease, HIV infection, malignancy, smoking, and alcohol use. Clinical features at presentation, such as fever, cough, dyspnoea, shock, altered sensorium, icterus, diarrhoea, extrapulmonary symptoms, lymphadenopathy, and focal neurological deficits, were documented. Radiological findings were classified as unilateral or bilateral infiltrates. Laboratory parameters, including complete blood counts, renal and liver function tests, and C-reactive protein (CRP) levels, were also reviewed and categorized according to standard clinical thresholds where possible. Microbiological data included results of blood and respiratory cultures and detailed antimicrobial susceptibility profiles, with a focus on resistance to ceftriaxone, piperacillin–tazobactam, carbapenems, and fluoroquinolones. Isolates were identified using the VITEK Mass Spectrometry (MS) system based on matrix-assisted laser desorption ionization–time of flight mass spectrometry (MALDI-TOF MS; bioMérieux, France). Antimicrobial susceptibility testing was performed using the automated VITEK 2 system (bioMérieux, France), and results were interpreted according to the Clinical and Laboratory Standards Institute (CLSI) guidelines [11]. Isolates were categorized as susceptible or resistant based on the corresponding clinical breakpoints.

Statistical analysis was performed using both descriptive and inferential methods. Continuous variables were summarized as medians with interquartile ranges (IQRs) and compared between severe and non-severe groups using the Mann–Whitney *U* test. Categorical variables were expressed as proportions and compared using the Chi-square test. In the univariate analysis, variables with p-values <0.05 were included in the multivariable logistic regression model to identify severity-related factors. Variables with more than 20% missing data were excluded to minimize bias. When collinearity was suspected, the simpler, more clinically feasible variable was selected. Model building followed the principle of parsimony, with stepwise removal of non-contributory variables to derive a stable final model. Adjusted odds ratios (aORs) with 95% confidence intervals (CIs) were reported, and model calibration was assessed using the Hosmer–Lemeshow goodness-of-fit test. A two-tailed p-value of <0.05 was considered statistically significant.

## 3 Results

During the seven-year study period, 277 culture-confirmed cases of *K. pneumoniae* were identified, and the samples were sent within 48 h of admission. After applying the predefined eligibility criteria, 105 patients were included in the final analysis. Exclusions were due to prolonged symptom duration beyond 14 days (n = 42), absence of new radiological infiltrates (n = 42), possible hospital-acquired pneumonia (n = 37), and polymicrobial infections (n = 51).

Among the 105 included patients, 32 (30.5%) had severe disease, while the remaining 73 (69.5%) had non-severe disease. There was only one patient in the entire cohort who died during the hospital stay. Patients with severe disease tended to be older (p = 0.097). Male predominance was consistent across both groups (p = 0.729) (Table 1). Diabetes mellitus was the most common comorbidity, present in approximately half of the cohort, with comparable rates between the severe and non-severe groups (53.1% vs. 47.9%, p = 0.625) (Table 1). In contrast, alcohol use was significantly more common in the severe group (21.9% vs. 4.1%, p = 0.004). Other comorbidities, including chronic kidney disease, chronic liver disease, chronic lung disease, malignancy, and smoking history, did not show significant differences between the groups (Table 1).

**Table 1:** Demographic characteristics and comorbidities of patients with *Klebsiella pneumoniae* CAP according to severity.

Parameter	Severe (n = 32)	Non-severe (n = 73)	p-value
Age (years), median (IQR)	64 (48.7–68.7)	57 (51.5–70.0)	0.097∗
Male gender, n (%)	23 (71.8)	50 (68.5)	0.729∗∗
Diabetes mellitus, n (%)	17 (53.1)	35 (47.9)	0.625∗∗
Alcohol use, n (%)	7 (21.9)	3 (4.1)	0.004∗∗
Malignancy, n (%)	3 (9.4)	5 (6.8)	0.653∗∗
HIV, n (%)	0 (0)	1 (1.4)	0.506∗∗
Chronic kidney disease, n (%)	5 (15.6)	6 (8.2)	0.254∗∗
Chronic liver disease, n (%)	2 (6.3)	2 (2.7)	0.387∗∗
Chronic lung disease, n (%)	9 (28.1)	26 (35.6)	0.454∗∗
Smoking history, n (%)	4 (12.5)	3 (4.1)	0.113∗∗

*Legend:* Mann–Whitney *U* test for continuous variables; Chi-square test for categorical variables.

CAP – community-acquired pneumonia; HIV – human immunodeficiency virus; IQR – interquartile range.

Clinical presentation was similar across groups, except for shock at presentation (Table 2). Altered sensorium, diarrhoea, extrapulmonary symptoms, and the distribution of radiological infiltrates (unilateral vs. bilateral) were not significantly different between severe and non-severe groups. Bacteraemia was more frequently documented among severe cases (15.6% vs. 2.7%, p = 0.015), highlighting systemic dissemination as a marker of disease severity.

**Table 2:** Clinical features of patients with *Klebsiella pneumoniae* CAP according to severity.

Clinical parameter		Severe (n = 32) n (%)	Non-severe (n = 73) n (%)	p-value
Icterus		1 (3.1)	0 (0)	0.129∗
Lymphadenopathy		0 (0)	1 (1.4)	0.506∗
Shock		4 (12.5)	0 (0)	0.002∗
Diarrhoea		2 (6.3)	1 (1.4)	0.167∗
Altered sensorium		1 (3.1)	1 (1.4)	0.545∗
Neck signs		0 (0)	1 (1.4)	0.506∗
Focal neurological deficit		1 (3.1)	2 (2.7)	0.913∗
Extrapulmonary symptoms		5 (15.6)	5 (6.8)	0.159∗
Bacteremia		5 (15.6)	2 (2.7)	0.015∗
Chest X-ray	Unilateral	24 (75.0)	57 (77.0)	0.729∗
	Bilateral	8 (25.0)	16 (21.9)	

*Legend:* Chi-square test was used.

CAP – community-acquired pneumonia.

Laboratory parameters at admission revealed distinct differences between groups. Median leukocyte counts were higher in severe cases (20.3 × 10^3^/μL) compared to non-severe cases (10.7 × 10^3^/μL, p = 0.008) (Table 3). Lymphocyte percentages were significantly lower in severe cases (6.4% vs. 15%, p = 0.008), consistent with severe systemic inflammation. Markers of organ dysfunction were also more deranged in the severe group, with higher serum urea (44 vs. 27 mg/dL, p = 0.001) and creatinine (1.11 vs. 0.87 mg/dL, p = 0.007). Hypoalbuminemia was notable, with severe cases having a mean serum albumin of 3.1 g/dL compared to 3.8 g/dL in the non-severe cases (p = 0.004) (Table 3). CRP levels were also significantly higher in severe cases patients (145 vs. 87 mg/L, p = 0.033). Among liver enzymes, aspartate transaminase (AST) was modestly but significantly elevated in severe (29.3 vs. 25 U/L, p = 0.038), while alanine transaminase (ALT) and bilirubin did not differ significantly between groups (Table 3).

**Table 3:** Laboratory parameters in patients with *Klebsiella pneumoniae* CAP by severity.

Laboratory parameters	Severe (n = 32)	Non-severe (n = 73)	p-value
CRP (mg/L)	145 (109–253)	87 (36–151)	0.033∗
WBC ( × 10^3^/μL)	20.3 (10.1–23.9)	10.7 (7.6–12.5)	0.008∗
Neutrophil %	80.6 (63.5–84.7)	68 (63–78)	0.392∗
Lymphocyte %	6.4 (2.5–8.1)	15 (7.5–19.7)	0.008∗
Urea (mg/dL)	44 (18–75.5)	27 (19–41)	0.001∗
Creatinine (mg/dL)	1.11 (0.74–1.8)	0.87 (0.71–1.22)	0.007∗
Total bilirubin (mg/dL)	0.47 (0.29–0.63)	0.51 (0.37–0.76)	0.198∗
AST (U/L)	29.3 (26.9–39.5)	25 (20.1–53)	0.038∗
ALT (U/L)	36.5 (18.5–54.8)	25 (21.0–50.5)	0.072∗
Albumin (g/dL)	3.1 (2.7–3.5)	3.8 (3.4–4.0)	0.004∗

*Legend:* Mann–Whitney *U* test was used.

ALT – alanine aminotransferase; AST – aspartate aminotransferase; CAP – community-acquired pneumonia; CRP – C-reactive protein; IQR – interquartile range; WBC – white blood cell count.

Microbiological analysis revealed substantial antimicrobial resistance, particularly to ceftriaxone (Table 4). Overall, 40% (42/105) of isolates were ceftriaxone resistant. Resistance was significantly more common in isolates from patients with severe disease (56.2% vs. 30.1%, p = 0.011). Resistance to piperacillin–tazobactam (12.5% vs. 6.3%, p = 0.047) and carbapenems (9.4% vs. 0%, p = 0.008) was also higher among severe cases. Fluoroquinolone resistance showed a non-significant trend toward greater frequency in the severe group (34.4% vs. 17.8%, p = 0.063).

**Table 4:** Antimicrobial resistance profile of *Klebsiella pneumoniae* isolates in CAP patients according to severity.

Antimicrobial resistance	Severe (n = 32) n (%)	Non-severe (n = 73) n (%)	p-value
Ceftriaxone resistance	18 (56.3)	22 (30.1)	0.011∗
Fluoroquinolone resistance	11 (34.4)	13 (17.8)	0.063∗
Piperacillin–tazobactam resistance	4 (12.5)	2 (6.3)	0.047∗
Carbapenem resistance	3 (9.4)	0 (0)	0.008∗

*Legend:* Chi-square test was used.

CAP – Community-acquired pneumonia.

On multivariable logistic regression analysis, four independent factors associated with severity were identified (Table 5). Ceftriaxone resistance was associated with severity, with an adjusted odds ratio of 6.6 (95% CI 2.1–20.5, p = 0.001). Alcohol use was associated with 18.8-fold higher odds of severe disease (95% CI 3.5–101, p = 0.001), while bacteremia was associated with 11.8-fold increased odds (95% CI 1.8–77.5, p = 0.01). Leukocytosis also remained a significant factor, with each unit increase in total leukocyte count associated with 15% higher odds of a severe disease (aOR 1.15, 95% CI 1.05–1.26, p = 0.002) (Table 5). The final model demonstrated excellent calibration, with a Hosmer–Lemeshow goodness-of-fit p-value of 0.934, confirming strong agreement between observed and predicted outcomes.

**Table 5:** Multivariable logistic regression analysis of predictors of severity in *Klebsiella pneumoniae* CAP.

Parameter	Unadjusted OR (95% CI)	Adjusted OR (95% CI)	p-value
Alcohol use	6.5 (1.6–27.2)	18.8 (3.5–101)	0.001
Bacteraemia	6.6 (1.2–35.9)	11.8 (1.8–77.5)	0.010
WBC count ( × 10^3^/μL)	1.12 (1.04–1.20)	1.15 (1.05–1.26)	0.002
Ceftriaxone resistance	2.98 (1.26–7.00)	6.6 (2.1–20.5)	0.001

*Legend:* Hosmer–Lemeshow test for goodness-of-fit: 0.934. Variables with >20% missing values were excluded (e.g., CRP). In cases of collinearity, the simpler and more routinely available variable was selected. The final model was derived using stepwise exclusion guided by the principle of parsimony.

CAP – community-acquired pneumonia; CI – confidence interval; OR – odds ratio; WBC – white blood cell count.

## 4 Discussion

In this seven-year retrospective cohort of culture-confirmed *K. pneumoniae* CAP, 105 patients met the eligibility criteria, of whom nearly one-third had severe disease. Severe cases were more frequently associated with alcohol use, bacteraemia, shock at presentation, leukocytosis, lymphopenia, renal dysfunction, hypoalbuminemia, and elevated inflammatory markers. Antimicrobial resistance was common, with 40% of isolates resistant to ceftriaxone, and resistance rates were significantly higher among severe cases. In the multivariable analysis, four factors remained independently associated with severity: alcohol use, bacteraemia, leukocytosis, and ceftriaxone resistance, and the final model demonstrated excellent calibration. These findings highlight the role of host factors, systemic dissemination, and antimicrobial resistance in influencing the severity of *K. pneumoniae* CAP.

Our results are consistent with a systematic review of Indian studies, which identified *K. pneumoniae* (23%) as the second leading cause of CAP. [2]. Notably, the review highlighted the rising contribution of *K. pneumoniae* to CAP, a trend echoed by individual reports across the country [[12], [13], [14]]. International studies from France, Taiwan, Nigeria, and Iraq further reinforce both the clinical significance of *K. pneumoniae* in CAP and the growing burden of resistance [[3], [4], [5],[15], [16], [17]]. In our cohort, antimicrobial resistance was significantly higher among patients with severe disease across all agents tested. Although resistance to piperacillin–tazobactam and carbapenems was not very high, their detection suggests possible prior antibiotic exposure, a history that could not be systematically evaluated in this study. Fluoroquinolone resistance was also noted, and although some isolates remained susceptible, their use should be discouraged in tuberculosis-endemic regions [18]. For regression analysis, we selected ceftriaxone resistance as the primary variable to avoid collinearity with other β-lactam agents, as ceftriaxone is the most widely used empiric agent for CAP in our setting. The strong association between ceftriaxone resistance and severe disease in our cohort aligns with national and international evidence underscoring the inadequacy of standard ceftriaxone-based regimens in managing *K. pneumoniae* CAP.

Our finding that alcohol use, bacteraemia, and leukocytosis were strongly associated with severe disease aligns with existing literature [19,20]. Alcohol use is not only a biological risk factor but also frequently coexists with poor socioeconomic and nutritional status, both of which may further compromise host immunity and delay access to timely care. Biologically, alcohol impairs mucosal barriers, disrupts local airway defense mechanisms, and suppresses systemic immune responses, thereby predisposing individuals to severe Gram-negative infections. Bacteremia, in turn, reflects systemic dissemination of infection and is consistently associated with poor prognosis in CAP, serving as a robust marker of severity [20]. Leukocytosis, although nonspecific, emerged in our study as a simple, clinically accessible predictor of severe *K. pneumoniae* CAP, aligning with prior CAP studies that have leveraged readily available inflammatory markers for early risk stratification [19]. Together, these factors emphasize the importance of integrating host vulnerabilities and basic laboratory parameters into bedside decision-making for patients with *K. pneumoniae* CAP.

In regions where *Klebsiella pneumoniae* is common, and ceftriaxone susceptibility is low, empiric regimens designed primarily to target *Streptococcus pneumoniae* may be inadequate, particularly in severe CAP [8]. Reliance on ceftriaxone-based combinations risks undertreatment, potentially leading to delayed initiation of effective therapy, progression to septic shock, and increased mortality [8]. Our findings further demonstrate that ceftriaxone resistance itself was independently associated with severe disease, underscoring the clinical consequences of inadequate empiric coverage. These results support a risk-stratified approach that integrates local microbiological epidemiology, resistance patterns, and patient-specific risk factors such as alcohol use, diabetes, bacteremia, or marked leukocytosis. In patients presenting with these high-risk features or with severe disease at admission, empiric regimens providing broader Gram-negative coverage, such as piperacillin-tazobactam, where local susceptibility remains acceptable, or carbapenems in critically ill patients or settings with high ESBL prevalence, may be warranted pending microbiological confirmation. Conversely, lower-risk patients without such features may still be appropriately managed with standard CAP protocols. Importantly, any empiric escalation must be guided by institutional antibiogram data and accompanied by timely de-escalation based on culture and susceptibility results to preserve antimicrobial stewardship. Strengthening diagnostic capacity through rapid culture techniques, susceptibility testing, and emerging molecular diagnostics is crucial to facilitate early pathogen-directed therapy and improve outcomes. Ultimately, region-specific treatment guidelines that reflect the growing contribution of Gram-negative pathogens to CAP are needed, rather than relying predominantly on recommendations derived from Western cohorts dominated by *S. pneumoniae* [21].

Our study has several notable strengths. By restricting inclusion to culture-confirmed, monomicrobial cases of *K. pneumoniae* CAP, we minimized diagnostic uncertainty and avoided the confounding influence of polymicrobial infections common in respiratory cultures. This methodological rigor allowed for a clearer attribution of outcomes to *K. pneumoniae*. In addition, we employed a structured and comprehensive data extraction process from electronic health records, ensuring systematic capture of demographic, clinical, laboratory, and microbiological variables. The use of robust multivariable logistic regression analysis, with careful attention to collinearity and model parsimony, further strengthened the validity of our findings by identifying independent predictors of severity rather than relying solely on unadjusted associations. Our study also spanned seven years, providing a large temporal window.

Nevertheless, several limitations warrant acknowledgement. First, this was a single-centre retrospective study. While our hospital serves as a large referral centre in South India, the findings may not be fully generalizable to other regions with different patient populations, healthcare access, or antimicrobial resistance profiles. Second, as with all retrospective analyses, residual confounding and missing data cannot be entirely excluded, despite efforts to mitigate bias through exclusion criteria and variable selection. Third, we did not characterize molecular resistance mechanisms (e.g., extended-spectrum β-lactamases or carbapenemase genes) or assess the presence of hypervirulent strains. This could have provided additional insight into the interplay between resistance and virulence in driving clinical outcomes. Fourth, we used ICU admission as a pragmatic severity endpoint. Although international guidelines provide standardized definitions of severe CAP, complete data required to reconstruct these scores were not uniformly available in this retrospective dataset. ICU admission was therefore selected as an objectively documented and clinically meaningful surrogate marker that reflects real-world decision-making and resource utilization, particularly in low- and middle-income settings. Nevertheless, this definition does not strictly align with guideline-based severity criteria and may have been influenced by institutional admission practices and bed availability. Fifth, the observed in-hospital mortality was low, and mortality was assessed only for deaths occurring during the index hospitalization. Post-discharge mortality and 30-day outcomes were not available. In addition, inclusion required the retrieval of samples for culture confirmation of *Klebsiella pneumoniae* within 48 h of admission, meaning that only patients who survived long enough for microbiological sampling and confirmation were captured. Patients who deteriorated rapidly and died before culture acquisition or confirmation would not have been included, introducing the possibility of immortal time bias and potentially leading to underestimation of true mortality. Lastly, the role of previous antimicrobial exposure or empirical antimicrobial therapy could not be directly assessed. Although national guidelines and hospital policy recommend ceftriaxone in combination with azithromycin or doxycycline, the retrospective nature of this study precluded reliable capture of the actual empiric regimens prescribed or their duration.

## 5 Conclusion

Our findings, in line with prior Indian and international reports, demonstrate that *K. pneumoniae* CAP is associated with substantial severity, driven by host factors, systemic dissemination, and antimicrobial resistance. Alcohol use, bacteraemia, leukocytosis, and ceftriaxone resistance emerged as independent correlates of severe disease, underscoring the importance of both patient-related and microbiological determinants. The high rate of ceftriaxone resistance underscores the potential inadequacy of standard empiric regimens such as ceftriaxone–azithromycin, highlighting the need for region-specific empiric strategies guided by local resistance data. Incorporating early risk stratification, strengthening antimicrobial stewardship, and improving access to rapid diagnostics will be critical to optimizing management and improving outcomes in patients with *K. pneumoniae* CAP.

## CRediT authorship contribution statement

**Souvik Chaudhuri:** Writing – original draft, Methodology, Data curation. **Tirlangi Praveen Kumar:** Writing – review & editing, Writing – original draft, Data curation. **Shreya Das Adhikari:** Writing – review & editing, Writing – original draft, Methodology, Data curation. **Danavath Nagendra:** Writing – review & editing, Writing – original draft, Data curation. **Prithvishree Ravindra:** Writing – review & editing, Writing – original draft. **Rachana Bhat:** Writing – review & editing, Writing – original draft. **K.E. Vandana:** Writing – review & editing, Writing – original draft, Methodology. **Nitin Gupta:** Writing – review & editing, Writing – original draft, Supervision, Project administration, Methodology, Investigation, Formal analysis, Data curation.

## Consent to participate

Because this study used a retrospective design, informed consent was waived.

## Ethics approval

This study received ethical clearance from the Institutional Ethics Committee of Kasturba Medical College and Kasturba Hospital (IEC1:557/2025), in compliance with the Declaration of Helsinki.

## Funding

We did not receive any funding for this study.

## Declaration of competing interest

The authors declare that they have no known competing financial interests or personal relationships that could have appeared to influence the work reported in this paper.

## Data Availability

Data will be available from the corresponding author upon reasonable request.
